# ‘He Never Liked Sport Anyway’ - Mother's Views of Young People Coping With a Bone Tumour in the Lower Limb

**DOI:** 10.1080/13577140500043823

**Published:** 2005

**Authors:** Emily A. Earle, Christine Eiser, Robert Grimer

**Affiliations:** 1 Department of Psychology University of Sheffield Sheffield S10 2TP UK; 2 Royal Orthopaedic Hospital Woodlands Birmingham B31 2AP UK

## Abstract

*Purpose:* Treatment for a bone tumour can compromise quality of life (QOL), especially for young patients. We used qualitative methods to assess mothers' views of patients' experiences and their coping strategies at approximately 6 months after diagnosis (T1: *n*=12) and 12–18 months later (T2: *n*=11).

*Subjects:* Mothers of young people (aged 6–22 years) who were undergoing treatment for either osteosarcoma or Ewing's sarcoma in the lower limb took part.

*Methods:* A semi-structured interview was devised to assess participation in sport, social life, schooling and general mobility. Interviews were transcribed and analysed using content analysis.

*Results:* Mothers reported a number of situations in which QOL was compromised, and these remained relatively constant over time (mean=4.25 at T1 and 4.27 at T2). However, strategies to manage these difficulties changed from Problem (constructive attempts to deal with challenges) to Emotion (managing the situation through use of emotions) focused coping from T1 to T2.

*Discussion:* Although the sample size is small, our results suggest that patients adopt a variety of coping strategies to deal with the physical and social restrictions associated with disease and treatment. The findings suggest that young people continue to experience many stresses up to 18 months after diagnosis. The shift from Problem to Emotion focussed coping over time may suggest a degree of acceptance of practical problems.


*Sarcoma*, March/June 2005; 9(1/2): 7–13

Taylor & Francis

Taylor & Francis Group

**ORIGINAL ARTICLE**

### 'He never liked sport anyway' – Mother's views of young people coping with a bone tumour in the lower limb

EMILY A. EARLE[1], CHRISTINE EISER[1], & ROBERT GRIMER[2]

[1]*Department of Psychology, University of Sheffield, Sheffield S10 2TP, UK, and* [2]*Royal Orthopaedic Hospital, Woodlands, Birmingham B31 2AP, UK*

**Abstract**

*Purpose*: Treatment for a bone tumour can compromise quality of life (QOL), especially for young patients. We used qualitative methods to assess mothers' views of patients' experiences and their coping strategies at approximately 6 months after diagnosis (T1: $n = 12$) and 12–18 months later (T2: $n = 11$).

*Subjects*: Mothers of young people (aged 6–22 years) who were undergoing treatment for either osteosarcoma or Ewing's sarcoma in the lower limb took part.

*Methods*: A semi-structured interview was devised to assess participation in sport, social life, schooling and general mobility. Interviews were transcribed and analysed using content analysis.

*Results*: Mothers reported a number of situations in which QOL was compromised, and these remained relatively constant over time (mean = 4.25 at T1 and 4.27 at T2). However, strategies to manage these difficulties changed from Problem (constructive attempts to deal with challenges) to Emotion (managing the situation through use of emotions) focused coping from T1 to T2.

*Discussion*: Although the sample size is small, our results suggest that patients adopt a variety of coping strategies to deal with the physical and social restrictions associated with disease and treatment. The findings suggest that young people continue to experience many stresses up to 18 months after diagnosis. The shift from Problem to Emotion focussed coping over time may suggest a degree of acceptance of practical problems.

**Keywords:** *Osteosarcoma, ewing's tumour, young people, coping, QOL*

### Introduction

Survival rates following bone cancer in childhood have improved substantially since the 1970s and now approach 65% [1]. Although primary amputation is sometimes necessary, limb salvage surgery (LSS) remains the treatment of choice. Disadvantages of LSS include increased risk of infection, breakages and regular hospitalisations in younger patients to lengthen the prosthesis. For patients, quality of life (QOL) is potentially compromised by the restrictions to mobility associated with the prosthesis. In addition, they are vulnerable to a number of late-effects associated with chemotherapy. Thus, survivors are vulnerable to physical, social and behavioural late-effects as a result of the disease and treatment.

Increasingly, it is acknowledged that evaluation of the efficacy of any treatment must take into account not only survival and morbidity, but also QOL. In the context of health and illness, QOL has been defined as the subjective and objective impact of dysfunction linked with an illness, injury, or medical treatment [2]. Previous work has focused on differences in QOL following amputation or LSS, in both adult [3,4] and younger patients [5,6].

QOL has also been defined in terms of a balance between what an individual can, and would like to be able to do. 'Quality of life measures the difference, or the gap, at a particular period of time, between the hopes and expectations of the individual and the individual's experience. It is concerned with the difference between perceived goals and actual goals. It is an assessment of the potential for growth' (Ref. 7, p. 184). Thus, a good QOL occurs when individual's experiences are matched by their hopes and ambitions Diagnosis of a bone tumour during

Correspondence: Professor Christine Eiser, Cancer Research UK Child and Family Research Group, Department of Psychology, University of Sheffield, Western Bank, Sheffield S10 2TP, UK. Fax: +44-114-276-6515. E-mail: c.eiser@sheffield.ac.uk

ISSN 1357–714X print/ISSN 1369–1643 online © 2005 Taylor & Francis Group Ltd
DOI: 10.1080/13577140500043823

childhood or adolescence will necessarily challenge the likelihood of matching daily reality with hopes and ambitions.

In managing or coping with such gaps or discrepancies, two general approaches have been identified [8]. *Problem-focused* coping involves constructive attempts to reduce the harm, threat or challenge of a situation, and has been contrasted with *Emotion-focused* coping, which involves efforts to manage the situation through regulation of emotions. Problem-focused coping is typically employed in situations where something constructive can be done and may be more beneficial to general well being [9]. However Emotion-focused coping is often the only option when dealing with health problems, especially where adverse situations cannot be changed [10]. As an example, young patients may find that they are unable to play contact sports following diagnosis. A problem-focused strategy might include finding some way to remain involved even though it was not possible to play in the team. Especially in the longer term, patients choosing this approach may umpire or coach junior teams. A more Emotion-focused strategy in contrast might involve changing views about the importance of sport, for example asserting that you never really enjoyed sport. Problem-focused coping emerges earlier in childhood than Emotion-focused coping, which is not generally observed until late childhood or adolescence (11).

The aims of this paper are, first, to describe the impact of a bone tumour and treatment on the everyday life of a small sample of young people, based on interviews with their mothers. Assessments are made at 6 months after diagnosis and 1 year later. Second, we describe QOL during treatment focusing on differences between what the individual wants and is able to do. Third, we describe the strategies adopted to resolve discrepancies and achieve a more acceptable QOL. The initial plan was to recruit families within 6 months of diagnosis. However, there was considerable variability in patient's health at this time, with the result that recruitment took place over a period of 18 months following diagnosis. We therefore report the results in terms of two groups; those recruited within 6 months of diagnosis (T1) and those recruited within 12–18 months (T2). Seven mothers were seen on both occasions.

### Method

### *Sample*

Recruitment took place over an 18 month period. The sample included mothers of children and young people aged between 6 and 22 years who were diagnosed with either an osteosarcoma or Ewing's tumour in the lower limb. During the course of the study, 16 mothers agreed to take part. Twelve mothers were recruited within 6 months of diagnosis and seen at T1. Of these, two patients died and three were too ill to be seen again at T2. Therefore only seven were seen at both time points. Four additional mothers were recruited within 12–18 months of diagnosis and were seen once only at T2. The refusal rate was approximately 50%.

Demographic data is shown in Table I.

Table I. Demographic data of the patients of whose mother was interviewed.

| | T1 ($n=12$) | T2 ($n=11$) |
|---|---|---|
| Age range (years) | 6–19 | 7–22 |
| Mean age (years) | 12 | 14 |
| No. of females | 3 | 3 |
| No. of males | 9 | 8 |

### *Procedure*

Approval to conduct the study was obtained from the appropriate Ethics Committee. Mothers were given verbal and written information by a research nurse who gained signed consent. Mothers who agreed were then contacted by telephone by a member of the research team who subsequently visited them at home. Patients and fathers were occasionally present at interviews; however, this was rare and their contribution to the interview was invariably limited. For this reason, their data were not used in analysis. Twelve mothers were interviewed at T1, and eleven mothers at T2.

### *Interview*

A semi-structured interview schedule was devised to focus on themes identified from previous research as relevant to QOL of the patients. Themes included:

1. Sport and physical activities.
2. School: absences, effect on work, concentration.
3. Social and family: relationships with peers, friends and family.
4. Appearance: hair loss, scars, weight loss/gain.
5. Mobility: walking, self-care, going out, independence.
6. Emotional/understanding: emotional effects, understanding of illness.

The interview lasted approximately 90 min.

### Treatment of data

### *Coding of interviews*

Interviews were transcribed by a trained secretary. A content analysis was carried out on the data by a principal researcher with the aim of identifying all examples of discrepancies and strategies.

*Discrepancies*

A discrepancy was defined as any clear statement of a difference between what mothers reported a patient could, and would like to do in any aspect of their life. The discrepancies were further categorised according to the key themes defined in the interview.

*Strategies*

A strategy was defined as any attempt to reduce the difference between what mothers reported the patient could and would like to be able to do. Strategies were further coded as Problem- or Emotion-focused coping based on accepted definitions [7,8].

*Problem-focused coping*

1. Make plans and set targets; devise a realistic achievable plan that will help compensate for an activity that is no longer achievable (e.g., within a couple of hours he'd sort of accepted it and said 'well, if that's the case and I can't do the sports, particularly football which he was very interested in, I'll do something else').
2. Change activity; substitute an unachievable activity with another or change a preferred activity (e.g., 'he can't actually play football .... he started going to snooker club').

*Emotion-focused coping*

1. Denial/acceptance: no practical solution is identified but mothers report patients dealt with the discrepancy by denial or resignation (e.g., 'I think he's resigned to not playing cricket or hockey at any level again').
2. Optimism: the use of 'positive thinking'. Mothers report that patients are hopeful for the future and state that things will be okay (e.g., 'since that point he's been very positive .... apart from a very few occasions when he's been down.... He's maintained a very positive outlook').
3. Social comparison: patients deal with discrepancies by comparing themselves favourably with someone equally or less fortunate than themselves (e.g., 'he said...when he eventually started losing it (hair).... well at least I look like David Beckham now).
4. Seeking support from others: patients seek advice or social support from others (e.g., 'she gets lots of encouragement, like her exercises, we all used to take part in them, we'd all do them... just so that she wasn't on her own')

*Inter-rater reliability*

Fifty percent of the interviews were coded independently by a second researcher. Inter-rater reliability was high (96%), and any differences were resolved by discussion.

### Results

*Time 1*

General description. Mothers reported major disruption to their child's everyday school, work and social lives. Three patients were not attending full time education at all, and the rest had extended absences. Two patients had missed important GCSE exams and older patients ($n = 3$) were anxious about missing long periods away from college or university. All were unable to take part in sports activities and were generally inactive. Mothers reported many problems with friends and social activities, in that patients had very restricted opportunities for social contact, and found that friends were unable to support them or know what to say. Seven mothers reported problems associated with the prosthesis, pain or swelling in the leg. Patients had difficulties getting in and out of a bath, going up and down stairs, pain standing for short periods of time, and being unable to take part in school activities.

All mothers ($n = 12$) reported one or more discrepancies between what the patient wanted and was able to do (total = 51; mean = 4.25) (shown in Table II). In addition, they reported a total of 54 strategies to deal with the discrepancies. This is greater than the number of discrepancies because mothers occasionally reported more than one type of strategy to deal with a discrepancy. For the 54 strategies, 38 involved Problem-focused activities (change activity: 28; make plans: 10). The remainder ($n = 16$) involved Emotion-focused strategies (optimism: 8; denial/acceptance: 4; social support: 2; and social comparison: 2). For five reported discrepancies, no strategy was mentioned.

*Sport.* Not surprisingly, 11 of the 12 mothers reported a discrepancy in sports activities. The most common problem was inability to play contact sports ($n = 11$). Both Problem-focused ($n = 7$) and

Table II. Number of mothers reporting a discrepancy in each of the domains, for Time 1 and Time 2.

| Domain | T1 | T2 |
|---|---|---|
| Sport | 12 | 8 |
| Education | 11 | 8 |
| Social | 8 | 5 |
| Appearance | 5 | 9 |
| Emotional/understanding | 8 | 10 |
| Mobility | 7 | 7 |

Emotion-focused ($n = 6$) strategies were reported to deal with this. Problem-focused strategies included making plans ($n = 2$) 'his plans are to get back playing golf as soon as he can' and changing activities ($n = 5$) 'he started going to the snooker club' Families also reported Emotion-focused strategies ($n = 6$), including denial/acceptance ($n = 2$) 'it's not a huge problem'; optimism ($n = 3$) 'positive about being able to do it again' and social support ($n = 1$) 'I think they (past team members) have encouraged him to come and watch and generally help out'

*Education.* The same number of mothers ($n = 11$) reported discrepancies in achieving educational goals. These included being unable to return to school or college at all ($n = 3$), missing key appointments, and long absences. Discrepancies in this area were most often resolved through use of Problem-focused strategies ($n = 12$), in the form of making plans ($n = 5$) and changing activities ($n = 7$). These included arranging for exams to be taken at home, organising home tuition, re-taking a school year, and attending special catch up classes. There was no evidence of Emotion-focused strategies at this time.

*Social.* Discrepancies were reported by eight mothers, and included a lack of social contact, 'he really misses his old friends', and the problem of feeling uncomfortable around peers, 'she doesn't want her friend to see her when she is ill'. Problem-focused coping was again the more common strategy (n = 6). Parents organised transport so that patients could attend social functions and arranged for friends to visit. Emotion-focused coping was identified by only one mother, who was optimistic that opportunities for social activities would improve.

*Appearance.* Discrepancies concerning appearance ($n = 5$) included scarring – 'he has a long scar on his leg'; hair loss – 'she obviously lost her hair, it's hard for a little girl'; and weight loss – 'he lost a lot of weight during chemo'; 'he's more bony than he used to be' Discrepancies in this area were mainly resolved with Emotion-focused strategies ($n = 4$): denial/acceptance ($n = 2$) – 'the leg doesn't bother him'; 'he thinks it looks 'hard'; optimism ($n = 1$) – 'he knows his hair will come back'; social comparison ($n = 1$). Problem-focused coping ($n = 2$) was described where highly calorific foods were used in order to regain weight, and baggy trousers worn to disguise limping.

*Emotional/understanding of illness.* Discrepancies ($n = 8$) varied widely and included not being able to understand what was happening 'Doctors didn't have time to explain so that we understand'; becoming phobic 'he developed a phobia of needles' and fearful 'he may not be able to have children'. Both Problem- and Emotion-focused strategies were described. Problem-focused strategies included making plans ($n = 5$) 'we share responsibility for the tablets'; 'we keep a diary of what we have to do'. In terms of Emotion-focused strategies ($n = 4$), there were examples of optimism ($n = 2$) 'she is positive she will get stronger', social comparison ($n = 1$) 'there was a lad there who had been going back 12 years. He had the same thing and he explained everything' and social support ($n = 1$) 'help is offered when its needed'.

*Mobility.* Discrepancies ($n = 7$) included being unable to sit comfortably on a chair 'because of the tumour in his bottom'; unable to drive or carry things, problems standing, walking 'he can't walk in certain places because of the risk of tripping', and moving up and down stairs. Again discrepancies in this area were resolved predominantly with Problem-focused strategies ($n = 6$), including use of a wheelchair or crutches and changing the car – 'it is safer to drive an automatic because a fast movement on a brake may be restricting'. There was only one example of Emotion-focused coping – 'and now he can drive and yes (he's happy) just finding things that he can do' (optimism).

### *Time 2*

*General description.* All but one patient of school age had made an attempt to return to full time education. There were major concerns about this one young person who had not yet returned to school and a second young person who attended only for certain lessons. Two other mothers described major difficulties on return to school. There was evidence of general revisions of plans and redefining ambitions, including reducing the number of exams to be taken and rethinking career goals. Difficulties surrounding play time and rough playground activities were a common concern. Problems with physical appearance were prevalent, including concerns about attaining a normal weight, scarring and the wasted appearance of the affected leg. Mothers at this time were acknowledging the restricted nature of future functioning. Anxieties were expressed about the patient going out alone or using public transport, related to limited ability to run or move quickly. Mothers also looked back on their experiences and pointed to the lack of counselling and advice during the early stages of treatment, and distress associated in knowing other young people who had died. Anxieties about the future were also emerging, including concerns about concentration and poor memory, as well as physical late-effects including potential fertility problems.

A total of 47 discrepancies (mean = 4.27) were reported at T2 and are summarised by domain in Table II. Fifty-five strategies were reported to deal with the discrepancies. Twenty-four were Problem-focused (change activity: 21; make plans: 3). Thirty-one were Emotion-focused (optimism: 9; denial/acceptance: 21; social support: 1; and social comparison: 0).

*Sport.* Eight of the 11 mothers reported sports discrepancies, which included on-going concerns about the inability to play contact sports 'he couldn't play because of his bad knees .... and now he's been told he might not be able to lift .... and running may be a problem'; 'he's not supposed to jar the prosthesis in the top of the thigh bone'. At T2, they were resolved predominantly with Emotion-focused strategies ($n = 6$). These included denial/acceptance ($n = 4$) 'he's never indicated that he misses playing football'; and optimism ($n = 2$) – 'I'm hoping that reasonably soon things are going to get back to normal'. In terms of Problem-focused strategies ($n = 4$), two families had changed their activities – 'he's a tremendous young cricket coach', and two had made plans to do so 'I'm trying to get them a few riding lessons'.

*Education.* At T2, emphasis was on the return to education and catching up with missed work, or reducing the number of exams taken ($n = 8$). Two mothers reported that their son or daughter found it hard to settle back in 'she hasn't been to school yet and I don't think she will ever fit back in' Problem-focused strategies ($n = 5$) mainly involved extra help 'they put him in a foundation group for the kids who are a little bit slower'; 'he gets a tutor three times a week' and 'school didn't work so we're home schooling again'.

Emotion-focused strategies ($n = 6$) included denial/acceptance ($n = 4$) – 'we're fortunate that he's quite young' (doesn't do exams yet); and optimism ($n = 2$) – 'I mean he's relatively confident he's going to do well enough to go into sixth from next year and go into A-Levels next year'.

*Social.* Social discrepancies ($n = 5$) included loneliness 'he's lonelier in a crowd than he is in his own company'; not fitting in 'he finds a lot of his peers frankly a bit immature'; and difficulty seeing friends who are busy with GCSEs. Discrepancies were managed with a mix of Problem-focused strategies including making plans ($n = 1$) 'by going back to school of course he's seeing them more'; and changing activities ($n = 2$) 'there's a family we see pretty much every weekend'. Mothers also reported finding new ways to keep the patient in touch with friends 'he keeps in contact with them over the phone or internet'. Emotion-focused strategies included only optimism ($n = 2$) – 'he's a bit more understanding in the way that people feel'; 'he's getting more brave about talking to people instead of taking a back seat and so on'.

*Appearance.* Appearance was mentioned by nine of the mothers at T2. Discrepancies mentioned were similar to T1, including hair loss, weight loss, scarring and the appearance of the leg in general. Typically mothers reported Emotion-focused strategies ($n = 7$), all of which included elements of denial/acceptance 'I think he's quite proud of his scar to be honest. If you've gone through what he's gone through, you want to have something to show for it'. Problem-focused coping ($n = 3$) included wearing a hat or bandana, cutting hair short and having high calorie 'feeds' in hospital to combat weight loss.

*Emotional/understanding.* Discrepancies in this domain were mentioned by almost every mother ($n = 10$). These included a lack of sufficient follow-up care 'the after-support is just not there', counselling 'even if it was just a booklet of advice'; 'all cancer patients should get counselling as a matter of course'; problems with flashbacks and intrusions of painful treatment, difficulties coping emotionally such as fears about going out alone or exposure to germs, issues with fertility, pain and dealing with the deaths of other young people. Problem-focused coping ($n = 2$) included 'he's rediscovered a lot of interests ... he has a lot of projects that he wants to do'; and 'he has had a psychologist teaching him relaxation techniques'. Use of Emotion-focused strategies ($n = 6$) included denial/acceptance ($n = 4$) 'he tells me he doesn't want children anyway'; 'the more time goes on, the more he seems to be getting back to normal', optimism ($n = 1$) 'his attitude to life and his determination have come to the forefront' and social support ($n = 1$) 'she gets lots of encouragement'.

*Mobility.* Families ($n = 7$) were confronting the possible chronic nature of the restrictions that can be imposed on independence and mobility such as stiffness 'in the cold it freezes up', muscle wastage 'he didn't use his leg for such a long time it just wasted away', poor balance and pain 'it swells behind the knee'. Discrepancies were resolved using Problem-focused strategies ($n = 7$) and at T2 all were engaging in a new activity; or had found ways to take part in some physical activity 'I always have a rest before playing?'. In terms of Emotion-focused strategies ($n = 4$), families reported denial/acceptance ($n = 2$) 'his mobility is certainly not as good as it was before but he's well on the way to getting back towards that level', and optimism ($n = 2$) 'when they went back to school in January he said he wanted to walk'.

### Changes from time 1 to time 2

For the seven mothers who were interviewed at both T1 and T2, there were significantly more Problem-focused strategies at T1 (mean = 3.43) compared with T2 (mean = 2.14; $t = 2.46$, df = 6, $P < 0.05$). There was an increase in Emotion-focused coping from T1 (mean = 1.71) to T2 (mean = 3.28: $P = 0.07$), though this did not reach significance, possibly given the small sample size.

### Discussion

The diagnosis of a bone tumour during childhood and adolescence poses huge challenges to normal social, emotional and physical development. In this paper we have attempted to describe the particular challenges reported by a small group of mothers on two separate occasions.

As might be expected, concerns about schooling and education were prevalent at both times. For younger patients there are concerns about missing school and falling behind academically as well as worries about being knocked over in the playground. For older patients, there were concerns about being knocked over in the pub, being unable to take public exams on time, and the impact on work and career choices.

There was an overall shift from Problem- to Emotion-focused coping over time. This may be expected given that families may be unprepared on diagnosis for how long treatment may take and unaware of the range of setbacks that can be experienced. Following diagnosis, it may seem appropriate to make practical plans about getting back to normal life, but if these are difficult to achieve or unsuccessful, families may have to rethink their approach and adopt more Emotion-focused strategies. Emotion-focused strategies may also reflect acceptance of the disease over time. From a counselling perspective, these data have important implications. Families will need support to continue with Problem-focused strategies especially where they experience setbacks early in treatment.

In terms of Emotion-focused coping, we found denial/acceptance or optimism were more prevalent than either social comparison or social support. Since our numbers are small it is not possible to determine if different strategies are used more commonly for different domains, or at different times. Social comparison processes have been shown to be valuable in promoting QOL for some patients with cancer [12]. Thus, patients may evaluate their own progress in comparison with similar others, and point to ways in which they cope with treatment more successfully than others. Where possible, this seems a very useful strategy, resulting in increased self-esteem. The adolescents in this sample were unlikely to know other patients with similar diseases, thus they would be forced to compare themselves with healthy friends, or themselves before they were ill. Both these comparisons would yield negative information, which possibly accounts for the small incidence of social comparison reported. Similarly, families consistently reported difficulties maintaining friendships with those they knew before the illness, discussing how living diverged and interests changed.

There are a number of limitations to our study. Although interviews were conducted on two separate occasions, the samples are small and only seven mothers were seen at both time points. Thus, this is not a truly longitudinal study. Small sample sizes are a consequence of the low incidence of these tumours and practice of treating patients for surgery in Supra-Regional Centres. Moreover, survival rates remain lower than other childhood cancers [13,14]. This reduced the number of mothers potentially available for interview at T2 and suggests that attention to physical care may seem more urgent than psychological care.

The small sample size meant that it was inappropriate to use standardized questionnaires and for this reason we adopted a qualitative methodology. This resulted in detailed information about the kind of difficulties patients experienced but did prevent us from comparing absolute QOL scores with previously published work. It has previously been shown that QOL of patients with cancer [15] and their mothers [16] is substantially below that expected from population norms based on standardized measures. In the future it would be important to determine any relationship between discrepancies, strategies and quality of life, but such an analysis is dependent on larger samples.

A further limitation relates to our reliance on mother's proxy reports of the young person's QOL and well-being. In view of the fact that only seven young people were willing to discuss their illness, we have focused this report on mothers' data. Given the age range (6–22 years) of patients in this study, we would expect most to be able to describe their own QOL, but many were unwilling or felt unable to discuss what were clearly difficult issues. In interpreting the results, it must be remembered that much previous work suggests mother's may over estimate difficulties compared with their children [17]. More comprehensive information is needed from children, since only they can directly report how their lives are affected in situations such as school, where mothers have no direct experience .

A final limitation involves the wide age range of patients in our study. It is well established that the impact of any chronic disease is age dependent [18]. Osteosarcoma and Ewing's sarcoma typically affects adolescents and young adults at a time when many decisions about work and adult social relations are made. Care of these patients involves some unique challenges compared with care of children and young people with other cancers. In contrast with other cancers, patients may experience multiple hospital admissions for limb lengthening and must be highly vigilant in order to minimise risks of breakage. Furthermore these difficulties extend into long-term survivorship [19]. Booklets and web-based information could prove invaluable in informing young people and their families about what to expect, including suggested strategies that others found helpful. Increasing awareness amongst health professionals of the difficulties these young people encounter when they finish treatment is a vital component of preparing them for school and social reintegration.

Our results suggest that the difficulties experienced are extensive, but adolescents show considerable resilience in their adoption of different coping strategies. These may be helpful in directing the content of more formal intervention programmes for those in need of professional support.

**Acknowledgement**

This work is funded by Cancer Research UK (C481/A121). We would like to thank Carol Hughes for recruiting patients to the study, and Yvonne Vance and Veronica Greco for help with data collection. We are grateful to all families who kindly gave up their time to take part.

**References**

1. Link M, Goorin AM, Horowitz M, et al. Adjuvant chemotherapy of high-grade osteosarcoma of the extremity. Updated results of the multi-institutional osteosarcoma study. *Clin Orthop* 1991;270:8–14.
2. Spieth LE, Harris CV. Assessment of health-related quality of life in children and adolescents: An integrative review. *J Ped Psychol* 1996;21(2):175–193.
3. Weddington WW, Segraves KB, Simon MA. Psychological outcome of extremity sarcoma survivors undergoing amputation or limb salvage. *J Clin Oncol* 1985;3:1393–1399.
4. Nagarajan R, Clohisy DR, Neglia JP, Yasui Y, Mitby PA, Sklar C, Finklestein JZ, Greenberg M, Reaman GH, Zeltzer L, Robison LL. Function and quality-of-life of survivors of pelvic and lower extremity osteosarcoma and Ewing's sarcoma: the Childhood Cancer Survivor Study. *Br J Cancer* 2004;91:1858–1865.
5. Eiser C, Darlington A-SE, Stride C, Grimer R. Quality of life implications as a consequence of surgery: limb salvage, primary and secondary amputation. *Sarcoma* 2001;5: 189–195.
6. Felder-Puig R, Formann AK, Mildner A, Bretschneider W, Bucher B, Zoubek A, Puig S, Topf R. Quality of life and psychosocial adjustment of young patients after treatment of bone cancer. *Cancer* 1998;83:69–75.
7. Calman KC. (1984). Quality of life in cancer patients- an hypothesis. *J Med Ethics* 1984;10:124–127.
8. Lazarus RS, Delongis A, Folkman S, Gruen R. Stress and adaptational outcomes; the problem of confounded measures. *Am Psychol* 1985;40(7):770–779.
9. Folkman S. Making the case for coping In: B. Carpenter, editor. *Personal coping: Theory, research and application*. New York. Praeger, 1992;31–46.
10. Vitalino PP, Mairo RD, Russo J, Katon W, Dewolfe D, Hall G. Coping profiles associated with psychiatric, physical health, work and family problems. *Health Psychol* 1990;9: 348–376.
11. Compas BE, Barnez GA, Malcarne V, Worsham N. Perceived control and coping with stress: A developmental perspective. *J Sociol Issues* 1991;47:23–24.
12. Buunk BP, Collons RL, Taylor SE, Van Yperen NW, Dakoff GA. The affective consequences of social comparison: Either direction has its ups and downs. *J Personality Social Psychol* 1994;59:1238–1249.
13. Stiller CA, Craft AW, Corazziari I. Survival of children with bone sarcoma in Europe since 1978: results from the EUROCARE study. *Eur J Cancer* 2001;37:760–766.
14. Terracini B, Coebergh J-W, Gatta G, Magnani C, Stiller C, Verdecchia A, et al. Childhood cancer survival in Europe: An overview. *Eur J Cancer* 2001;37(6):810–816.
15. Eiser C, Greco V, Vance YH, Horne B, Glaser A. Perceived discrepancies and their resolution: Quality of life in survivors of childhood cancer. *Psychol Health* 2004;19:5–28.
16. Sawyer M, Antoniou G, Toogood I, Rice M. A comparison of parent and adolescent reports describing the health-related quality of life of adolescents treated for cancer. *Int J Cancer (Suppl)* 1999;12:39–45.
17. Eiser C, Morse R. Quality-of-life measures in chronic diseases of childhood. *Health Technol Assess* 2001;5(4):1–156.
18. Kazak AE, Nachman GS. Family research on childhood chronic illness: Pediatric oncology as an example. *J Fam Psychol* 1991;4(4):462–483.
19. Hudson MM, Mertens, AC, Yasui Y, Hobbie W, Chen H, Gurney J, Yeazel M, Recklitis CJ, Neyessa M, Robison LR, Oeffinger KC. Health status of adult long-term survivors of childhood cancer: A report from the childhood cancer survivor study. *J Am Med Assoc* 2003;290:1583–1592.

